# Effects of Bathtub Bathing and Sauna Practices on Cardiovascular and Systemic Health: A Narrative Review

**DOI:** 10.3390/ijerph23030347

**Published:** 2026-03-10

**Authors:** Masayo Nagai, Akiko Tanaka

**Affiliations:** School of Nursing and Rehabilitation Sciences, Showa Medical University, Midiri-ku, Yokohama 226-8555, Japan; akiko-t@nr.showa-u.ac.jp

**Keywords:** bathtub bathing, thermal therapy, atherosclerotic cardiovascular disease, arterial stiffness, heat shock proteins, cardiovascular prevention, lifestyle

## Abstract

**Highlights:**

**Public health relevance—How does this work relate to a public health issue?**

- Daily bathing and sauna habits are associated with cardiovascular and metabolic indicators related to ASCVD risk.
- Thermal lifestyle practices represent modifiable, culturally embedded health behaviors with potential public health relevance.

**Public health significance—Why is this work of significance to public?**

- Evidence for sauna bathing is supported by long-term cohort studies, whereas evidence for habitual bathtub bathing remains limited despite its widespread daily use in Japan.
- Thermal exposure may influence inflammatory pathways involved in ASCVD pathophysiology, with potential relevance for public health.

**Public health implications—What are the key implications or messages for practitioners, policy makers and/or researchers in public health?**

- Safe and regular thermal bathing practices may support lifestyle approaches related to cardiovascular health.
- Further longitudinal and interventional public health research is needed to clarify causal mechanisms and guide policy and practice.

**Abstract:**

Thermal bathing practices, including domestic hot-water immersion and sauna use, have been linked to cardiovascular and systemic health outcomes. However, the amount and type of evidence differ between these practices. This review examines current epidemiological and mechanistic findings and discusses their relevance to cardiovascular health. A narrative review was conducted using Ichushi-Web and PubMed. Observational and interventional studies evaluating habitual bathtub bathing or sauna use in relation to physiological or health-related outcomes were included. Studies involving children or pregnant/postpartum women and those without relevant biological endpoints were excluded. In total, 45 studies met the inclusion criteria (17 on bathtub bathing and 28 on sauna use). Studies of habitual bathtub bathing, conducted mainly in Japan, have reported associations with lower arterial stiffness, improved glycemic control, and selected inflammatory or stress-related markers. Experimental work shows that hot-water immersion increases core body temperature, promotes peripheral vasodilation, and induces heat shock protein expression. Sauna bathing, investigated primarily in Finnish prospective cohorts, has been associated with lower cardiovascular and all-cause mortality, as well as favorable inflammatory and endothelial markers. Bathing conditions, age, sex, and cultural context appear to influence these findings. Thermal exposure produces circulatory and molecular responses relevant to cardiovascular regulation. Prospective data support associations between frequent sauna use and long-term outcomes, whereas evidence for domestic bathtub bathing is limited to observational studies of intermediate markers. Further research with clearly defined exposure parameters and clinical endpoints is needed to better understand the long-term cardiovascular implications of habitual thermal practices.

## 1. Introduction

In recent years, the effects described below have been increasingly examined in scientific research. Regular bathing and sauna use have been associated with various cardiovascular and systemic outcomes, including hypertension, cardiovascular disease (CVD), stroke, and neurocognitive disorders, potentially through mechanisms such as autonomic nervous system modulation, improved vascular endothelial function, and reduced oxidative stress and inflammation [1,2]. Among these practices, daily household bathing has drawn increasing attention as a practical and easily implementable method of health management [3,4]. Miyata et al. [5] reported that bathing habits and hot spring use in Japan were associated with indices of arterial stiffness. Furthermore, a large-scale epidemiological survey involving approximately 6000 individuals by Goto et al. [6] demonstrated that habitual bathtub bathing and year-round use of hot spring facilities were linked to better self-rated health.

The physiological mechanisms associated with bathing include vasodilation and enhanced blood flow due to thermal effects; increased central blood volume and secretion of atrial natriuretic peptide (ANP) because of hydrostatic pressure, along with improved respiratory function due to greater respiratory muscle load; and reduced musculoskeletal strain caused by buoyancy [7]. Collectively, these mechanisms are biologically relevant to circulatory dynamics and metabolic regulation, although their long-term clinical implications remain to be fully clarified.

Our previous research has also suggested a potential relevance of thermal practices to pathways involved in atherosclerotic cardiovascular disease (ASCVD) [8]. A review by Cullen et al. [9] indicated that passive heating may yield physiological responses comparable to some aspects of exercise, including improvements in cardiopulmonary function, vascular reactivity, glycemic control, and chronic inflammation. Moreover, thermal stimulation of skeletal muscle has been reported to enhance insulin sensitivity and improve glucose metabolism, highlighting its possible role as an adjunctive strategy in metabolic regulation [10]. In our own basic research, we observed increased glucose uptake and elevated expression of anti-atherogenic factors in skeletal muscle cells following thermal stimulation [11]. Thermal stimulation has also been proposed to affect cardiovascular function through the induction of heat shock proteins (HSPs). For instance, a review on the cardiovascular benefits of passive heat therapy noted that both acute and chronic upregulation of HSPs may promote nitric oxide-mediated vasodilation while attenuating oxidative stress and inflammatory responses [4]. Epidemiological data from Japan have further shown that a higher frequency of habitual bathing is associated with lower brachial-ankle pulse wave velocity (baPWV) and lower concentrations of B-type natriuretic peptide (BNP) [12]. Additionally, Kamioka et al. [13] reported that, among middle-aged and older individuals with diabetes, frequent home bathing was associated with better glycemic control. In our study involving young adults, habitual bathtub bathing was also associated with lower interleukin-6 (IL-6) levels, and bathing conditions such as duration and temperature appeared to influence inflammation-related biomarkers [14].

However, the overall health effects of thermal practices remain inconclusive. One key limitation is the considerable variety of study designs, including water temperature, humidity, bathing method, frequency, and duration, which complicate cross-study comparisons [15]. In addition, the available evidence differs in strength between sauna bathing and domestic bathtub bathing, and studies range from experimental investigations of intermediate biomarkers to observational research examining clinical outcomes. Careful differentiation of evidence levels is therefore necessary to avoid overinterpretation.

Overall, current findings suggest that bathing-centered thermal practices are associated with several physiological and health-related parameters relevant to circulatory and metabolic function. Nonetheless, the long-term effects of bathtub bathing remain insufficiently understood [12], and research explicitly addressing its relationship with clinical ASCVD outcomes is still limited.

The present study, therefore, aims to synthesize existing evidence regarding bathing habits and their relevance to vascular, metabolic, and inflammatory pathways related to cardiovascular health. In addition, because several studies retrieved during the literature search used sauna bathing as a primary form of thermal practice—particularly in non-Japanese populations—this review also organizes findings from sauna-related research while distinguishing differences in evidentiary strength between sauna and bathtub bathing.

In this review, habitual bathtub bathing refers to routine domestic immersion as practiced in daily life, whereas hot-water immersion describes structured interventions conducted under controlled conditions. Sauna bathing is treated as a distinct form of heat exposure.

## 2. Materials and Methods

This study is a narrative review summarizing previous research on bathing habits. The review was conducted in accordance with the general principles of transparent reporting for narrative reviews; however, it was not designed as a systematic review or meta-analysis.

During the screening process, several studies focusing on sauna use were also retrieved—particularly in overseas literature where sauna bathing is sometimes discussed within the broader context of bathing habits—and these studies were included when they met the eligibility criteria.

### 2.1. Literature Search

A literature search was initially conducted on 14 February 2025, using Ichushi-Web and PubMed. The search was expanded and updated on 22 February 2026, which was considered the final search date for this review. In Ichushi-Web, the search term “nyuyoku shukan” (bathing habits) was used and limited to human studies; conference proceedings were excluded. In PubMed, the search strategy was expanded to include the following terms: (“bathing habits” OR “hot water immersion” OR “sauna bathing”), with limits applied to human studies and English-language publications. No time restrictions were imposed.

### 2.2. Inclusion Criteria

Eligible articles were original research papers (observational or interventional) that examined: (i) habitual bathtub bathing or (ii) sauna use in the context of regular daily life, in relation to physical or health-related outcomes. Both observational studies assessing routine bathing habits and intervention studies were considered, provided that the exposure involved repeated bathing or sauna sessions rather than a single experimental heat exposure. We included studies that reported cardiovascular, metabolic, or inflammatory indicators relevant to cardiometabolic health, as well as studies evaluating long-term outcomes such as cardiovascular events or mortality.

### 2.3. Exclusion Criteria

Studies were excluded if they: (i) involved children or pregnant/postpartum women; (ii) were reviews or other secondary publications; (iii) did not report health-related outcomes; (iv) focused solely on the mineral composition of hot springs; or (v) did not clearly specify the bathing modality. Studies examining only a single, short-term heat exposure without repeated sessions were excluded. Heating procedures that did not correspond to recognizable bathtub bathing or sauna use in daily life (e.g., localized limb heating or device-based thermal stimulation) were not included. Studies limited to narrowly defined laboratory measures without accompanying cardiovascular or metabolic indicators were excluded.

## 3. Results

**Figure 1:**
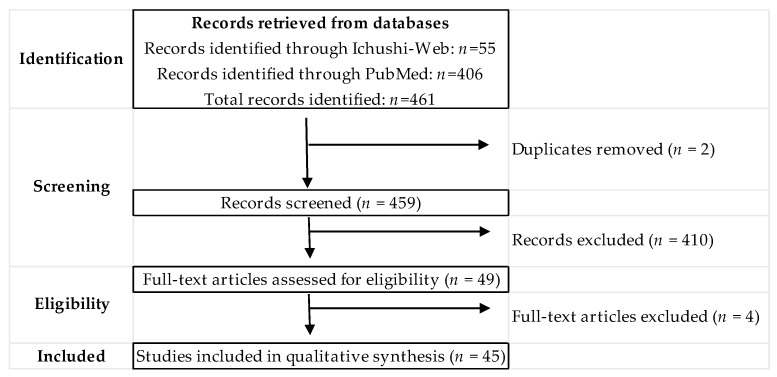
Study selection flow diagram. A total of 461 records were identified through database searching (PubMed, *n* = 406; Ichushi-Web, *n* = 55). After removal of duplicates (*n* = 2), 459 records were screened by title and abstract, and 410 records were excluded. 49 articles were assessed for eligibility, and 4 were excluded after full-text review. Finally, 45 studies were included in the qualitative synthesis, comprising 17 bathtub bathing studies and 28 sauna studies. Ichushi-Web (Igaku Chuo Zasshi) was searched as a primary source of Japanese medical literature.

A total of 461 records were identified through searches in Ichushi-Web (*n* = 55) and PubMed (*n* = 406). After removal of two duplicates, 459 records were screened, and 49 articles underwent full-text assessment. Ultimately, 45 studies were included in the qualitative synthesis (Figure 1).

Of these, 17 studies investigated bathtub bathing (including habitual domestic bathing and structured hot-water immersion interventions), and 28 examined sauna use.

### 3.1. Study Characteristics

The included studies addressed habitual bathtub bathing, experimental hot-water immersion interventions, and sauna use (Table 1 and Table 2).

Research on habitual bathtub bathing has mainly relied on observational designs, particularly cross-sectional studies conducted in Japan. These studies primarily assessed vascular indices, blood pressure, sleep parameters, and subjective health outcomes.

In addition, several recent studies from the United Kingdom and New Zealand examined structured hot-water immersion as an intervention. These experimental studies evaluated short-term cardiometabolic and vascular responses under controlled conditions.

By comparison, much of the sauna literature has been based on prospective cohort analyses. Many of these reports were derived from the Kuopio Ischaemic Heart Disease Risk Factor Study (KIHD), and several publications represent different outcome analyses from the same study population.

### 3.2. Bathtub Bathing Habits

Studies on habitual bathtub bathing were conducted mainly in Japan and were largely observational in design. Cross-sectional analyses were the most common approach [5,7,16,17,18,20], with a smaller number of longitudinal studies [12,19].

Reported outcomes focused primarily on surrogate cardiovascular measures and related physiological parameters. In several studies, more frequent bathing was associated with lower indices of arterial stiffness, including CAVI and baPWV, as well as lower central pulse pressure and BNP levels [5,7,12]. Higher bathing temperatures were also reported to be associated with more favorable vascular indices in some cohorts of older adults [7,12]. Beyond vascular measures, associations were described for sleep latency, subjective health, and mood [18,19], although findings were not entirely consistent across studies [5,16]. No studies evaluated hard cardiovascular endpoints such as incident cardiovascular events or mortality.

### 3.3. Hot-Water Immersion Interventions

In addition to observational studies of bathing habits, several interventional studies examined structured hot-water immersion (HWI) under controlled conditions in Japan, the United Kingdom, and New Zealand [21,22,23,24,25,26,27,28,29]. These studies primarily evaluated short-term physiological responses to repeated or acute heat exposure. Reported outcomes included increases in core body temperature, changes in stress-related or heat-shock protein markers, reductions in blood pressure, and alterations in metabolic parameters such as fasting glucose or insulin [21,22,23,24,25,26,27,28,29]. Sample sizes were generally small, protocols varied substantially, and outcomes were limited to intermediate physiological measures. No studies evaluated incident cardiovascular events or mortality.

### 3.4. Sauna Bathing

Studies on sauna bathing have been predominantly based on prospective cohort analyses, many of which were derived from the Kuopio Ischaemic Heart Disease Risk Factor Study (KIHD) [30,31,32,33,34,35,36,37,38,39,40,41,42,43,44,45,46,47,48,49]. Several publications represent different outcome analyses from the same underlying cohort., and reported outcomes varied across studies. Additional evidence has been reported from smaller cohort studies, clinical interventions, and cross-sectional surveys conducted in Finland and other countries, including supervised low-temperature sauna protocols in clinical populations [50,51,52,53,54,55,56,57].

Within the KIHD cohort, higher sauna bathing frequency was associated with lower risks of major cardiovascular and mortality outcomes, as well as several cardiometabolic and inflammatory parameters [30,31,32,33,34,35,36,37,38,39,40,41,42,43,44,45,46,47,48,49]. Studies outside the KIHD cohort have reported changes in vascular function, blood pressure, physical performance, and self-reported health measures following sauna exposure, although findings were not consistent across all outcomes [50,51,52,53,54,55,56,57].

Overall, the sauna literature includes both intermediate physiological measures and long-term clinical outcomes, while a large proportion of the available evidence originates from a single cohort population.

## 4. Discussion

### 4.1. Principal Findings

This review examined evidence on habitual bathtub bathing and sauna bathing use in relation to vascular and cardiometabolic health.

Evidence for habitual bathtub bathing is derived mainly from observational studies conducted in Japan and is largely limited to surrogate markers such as arterial stiffness, blood pressure-related indices, sleep parameters, and subjective health measures. Direct evidence linking household bathtub bathing to incident cardiovascular events remains unavailable.

In contrast, sauna bathing has been examined in prospective cohort studies, particularly in a Finnish population, where higher sauna frequency has been associated with lower risks of cardiovascular events and all-cause mortality. However, much of this evidence originates from a limited number of cohorts.

Overall, repeated thermal exposure appears to be associated with several physiological parameters relevant to cardiovascular risk. At present, however, the strength and type of evidence differ substantially between bathtub bathing and sauna bathing use.

### 4.2. Bathing Conditions and Thermal Load

The findings reviewed suggest that bathtub bathing frequency, timing, temperature, and duration collectively determine the magnitude of thermal load. However, these parameters vary considerably across studies, and standardized exposure definitions are lacking.

Most observational studies describe typical household bathtub bathing at approximately 39–41 °C for about 10–20 min [12,18]. Earlier Japanese surveys have reported similar bathing conditions [58]. However, some individuals report longer bathing durations exceeding 20 min [18,20]. Such thermal exposure can induce peripheral vasodilation and transient hemodynamic changes. In observational studies, frequent bathtub bathing (often defined as ≥5 times per week) has been associated with lower arterial stiffness indices and improved cardiometabolic markers in several cross-sectional analyses [5,7,12]. Evening bathing has also been linked to lower hypertension prevalence and improved sleep parameters [19,20]. Taken together, these observations suggest that habitual bathing practices may be associated with several physiological and behavioral factors relevant to cardiovascular health.

Nevertheless, most of these associations are derived from cross-sectional studies. Differences in lifestyle, physical activity, socioeconomic background, and health awareness may influence both bathtub bathing and health outcomes. In addition, reported bathing duration often refers to total time spent in the bathroom rather than precise immersion time, which complicates interpretation.

Thus, while bathing conditions appear to modulate physiological responses, current evidence does not allow identification of an optimal “dose” of bathing for cardiovascular prevention.

### 4.3. Physiological and Molecular Responses to Thermal Exposure

To clarify potential mechanisms, it is useful to distinguish acute physiological responses from cellular or molecular adaptations.

#### 4.3.1. Acute Circulatory and Autonomic Responses

Experimental studies have consistently demonstrated that hot-water immersion acutely increases core body temperature [21,22], induces peripheral vasodilation, and reduces arterial pressure [23]. Hydrostatic pressure during immersion increases venous return and stroke volume, potentially lowering total peripheral resistance and central blood pressure. Reductions in salivary amylase activity and improvements in mood states have also been reported [21,22], suggesting modulation of autonomic balance.

These acute responses provide a plausible explanation for short-term improvements in blood pressure and vascular function observed in small intervention studies.

#### 4.3.2. Heat Shock Proteins and Cellular Adaptation

Repeated thermal exposure has been associated with induction of heat shock proteins, particularly HSP70 [21]. Experimental cell studies have demonstrated altered expression of HSP family proteins and glucose metabolism-related genes following thermal stimulation [11]. Apoptosis-related gene networks under heat stress have also been reported [59]. Animal models further suggest potential renal protective effects under repeated mild heat stress [60].

HSP induction has been proposed to enhance nitric oxide-mediated vasodilation and attenuate oxidative stress and inflammatory signaling [4]. However, most supporting evidence derives from experimental or short-term studies, and direct links between HSP modulation and long-term ASCVD event reduction in humans remain unestablished.

#### 4.3.3. Inflammatory and Metabolic Markers

Associations between bathtub bathing habits and inflammatory markers have been inconsistently reported. Lower IL-6 levels were observed in young adults with habitual bathtub bathing [14], whereas effects on hsCRP have been less consistent. Improvements in glycemic control among patients with diabetes have also been described [13], although causal direction cannot be determined.

Overall, thermal exposure induces reproducible short-term circulatory and molecular responses. Whether repeated activation of these pathways translates into durable modification of atherosclerotic progression remains hypothetical.

### 4.4. Sauna Bathing: Cohort and Clinical Evidence

Sauna bathing has been examined more extensively than habitual bathtub bathing, particularly in prospective cohort studies conducted in Finland. A substantial proportion of this evidence is derived from the Kuopio Ischaemic Heart Disease Risk Factor Study (KIHD) [30,31,32,33,34,35,36,37,38,39,40,41,42,43,44,45,46,47,48,49], in which middle-aged men were followed longitudinally for cardiovascular and mortality outcomes.

Within the KIHD cohort, higher sauna frequency has been associated with lower risks of sudden cardiac death, coronary heart disease, stroke, cardiovascular mortality, and all-cause mortality [30,31,33,35,37,48,49]. Associations with inflammatory markers such as hsCRP, fibrinogen, and white blood cell count have also been reported [34,38,46]. In some analyses, sauna frequency appeared to modify the relationship between established risk factors and mortality, including systolic blood pressure and socioeconomic status [31,32,47]. However, these findings are based largely on repeated analyses of the same underlying population.

Evidence outside the KIHD cohort is more limited and based on smaller studies with diverse designs. Cross-sectional and survey-based investigations have reported associations between sauna use and subjective well-being or quality-of-life indicators [51,52,54]. Clinical and interventional studies conducted in selected patient populations, including individuals with chronic heart failure or peripheral artery disease, have documented improvements in exercise capacity, hemodynamic parameters, or endothelial function following repeated sauna exposure [53,56,57]. These studies provide useful physiological insights but involve relatively small samples and specific clinical contexts.

Sauna bathing is typically performed under relatively standardized temperature and humidity conditions, in contrast to the greater variability observed in household bathtub bathing. This relative consistency may facilitate exposure assessment in cohort settings. At the same time, sauna frequency may be influenced by socioeconomic status, lifestyle factors, and cultural norms [47], which should be considered when interpreting associations.

Overall, sauna bathing has been associated with both intermediate physiological markers and long-term cardiovascular outcomes in prospective analyses. Nevertheless, the concentration of outcome data within a single regional cohort and the observational nature of most studies indicate that findings should be interpreted with appropriate caution.

### 4.5. Age- and Sex-Related Differences

Bathtub bathing habits differ according to age and sex. Older adults tend to bathe more frequently and for longer durations, whereas younger individuals more often rely on showering rather than full tub immersion [16,61,62]. Women have been reported to use bathtubs more frequently than men [61].

Physiological responses to thermal exposure also vary by age. A significant negative correlation has been reported between age and the magnitude of core body temperature elevation during hot-water immersion [22]. Ishizawa et al. [22] estimated that the time required to achieve a relaxation-associated rise in body temperature was longer in adults aged ≥65 years than in those aged ≤44 years, likely reflecting age-related changes in body composition and circulating blood volume [63]. Experimental studies suggest age-related differences in IL-6 responses during hot-water immersion [64]. Cultural and lifestyle factors have also been associated with differences in inflammatory physiology in Japanese adults [65].

Sex-related differences in thermoregulatory responses have also been described. Under comparable bathtub bathing conditions, men exhibited greater increases in core body temperature than women [22], although the long-term clinical implications of this difference remain uncertain.

Sex-specific differences in cardiovascular risk profiles and preventive strategies have been highlighted in recent reviews of women’s cardiovascular health across the lifespan [66]. In this context, age- and sex-related variability should be considered when interpreting associations between thermal habits and cardiovascular outcomes.

### 4.6. Interpretation and Implications for ASCVD

Thermal exposure through bathing or sauna use induces acute physiological responses, including peripheral vasodilation, transient changes in blood pressure, and modulation of stress-related pathways [4,7,9]. Repeated mild heat stress has also been associated with alterations in heat shock protein expression and skeletal muscle-related metabolic pathways in experimental settings [11,67,68]. A recent review has noted that passive heating shares several physiological features with moderate-intensity aerobic exercise, particularly with respect to transient cardiovascular and vascular responses [9]. However, the magnitude and long-term clinical relevance of these similarities remain uncertain. Together, these observations provide a biologically plausible framework through which habitual thermal exposure could influence vascular and metabolic regulation.

Observational studies of bathtub bathing have reported associations with lower arterial stiffness indices [5,7,12], improved glycemic control [13], and reduced inflammatory markers in selected populations [14]. Similarly, sauna bathing in prospective cohorts has been associated with lower cardiovascular and all-cause mortality [30,31,32,33,34,35,36,37,38,39,40,41,42,48,49], as well as lower inflammatory marker levels [34,38,46]. Because arterial stiffness, chronic inflammation, and metabolic dysregulation are recognized contributors to ASCVD development, the observed associations are consistent with the hypothesis that habitual thermal exposure may influence cardiovascular risk profiles. However, direct evidence demonstrating that such physiological modulation translates into reduced ASCVD events is currently lacking.

At the same time, important distinctions must be maintained. For habitual bathtub bathing, evidence is largely observational and limited to intermediate markers; direct evaluation of incident ASCVD events has not been conducted. For sauna bathing, long-term outcome associations are available, but they are concentrated within a single regional cohort [30,31,32,33,34,35,36,37,38,39,40,41,42,43,44,45,46,47,48,49]. Moreover, both bathing and sauna frequency may correlate with broader lifestyle patterns and socioeconomic factors [47], which complicates causal interpretation.

Taken together, current evidence supports the biological plausibility that repeated thermal habits may contribute to cardiovascular risk modulation. However, the available evidence is insufficient to conclude that thermal habits prevent ASCVD. Prospective studies examining standardized exposure parameters and clinical endpoints across diverse populations will be necessary to clarify the role of thermal habits in long-term cardiovascular health.

### 4.7. Potential Role of Thermal Habits in ASCVD Prevention

Atherosclerotic cardiovascular disease develops over decades and is influenced by arterial stiffness, chronic inflammation, metabolic dysregulation, endothelial function, and blood pressure control. Several of these intermediate domains have been associated with bathtub bathing and sauna bathing practices in observational studies.

For sauna bathing, prospective cohort data—primarily from the KIHD study—demonstrate inverse associations between sauna frequency and cardiovascular and all-cause mortality [30,31,32,33,35,37]. These findings provide epidemiological evidence linking repeated heat exposure to long-term outcomes, although residual confounding cannot be excluded, and external validity is limited to similar populations.

In contrast, evidence for habitual bathtub bathing is largely limited to surrogate markers such as baPWV, CAVI, BNP, inflammatory indices, and glycemic control [5,7,12,13,14]. No prospective studies have evaluated incident ASCVD events in relation to bathtub bathing habits. Therefore, any inference regarding ASCVD prevention must be considered provisional.

From a mechanistic perspective, it is biologically plausible that repeated mild heat exposure may influence vascular tone, inflammatory signaling, autonomic balance, and metabolic regulation. However, the causal pathway linking acute physiological responses to long-term reduction in atherosclerotic events has not been demonstrated.

Accordingly, thermal bathing—particularly household bathtub bathing—should be regarded as a potentially health-supportive lifestyle behavior rather than an established preventive intervention for ASCVD. Further longitudinal and interventional studies with standardized exposure definitions are required to clarify whether the observed associations reflect causal effects.

Overall, current findings indicate that habitual thermal practices are associated with several vascular and metabolic parameters relevant to cardiovascular health. However, the available evidence differs between sauna bathing and household bathtub bathing, both in study design and in outcome assessment. Prospective data are largely limited to sauna cohorts, whereas evidence for bathtub bathing remains confined to intermediate markers. These distinctions should be kept in mind when considering the potential cardiovascular implications of habitual thermal exposure.

### 4.8. Safety Considerations

The potential health implications of bathtub bathing practices should be considered alongside safety evidence.

In Japan, bath-related accidents occur predominantly among older adults and increase during winter. Suzuki et al. (2019) described these incidents and suggested that circulatory instability during hot-water immersion may contribute in susceptible individuals, indicating possible acute risk in those with reduced cardiovascular reserve [69].

Sauna exposure also warrants contextual attention. Yang et al. (2018) reported elevated blood alcohol concentrations in many sauna-related deaths, suggesting that alcohol may act as an important modifying factor in heat-associated adverse events [70].

More broadly, passive heat therapy has been discussed as a cardiovascular stimulus; however, Rodrigues et al. (2024) emphasize the need for standardized protocols and appropriate risk assessment [71]. Thermal exposure induces circulatory and molecular responses, but their net effect likely varies by individual risk profile.

Furthermore, sauna-related cardiovascular findings appear embedded within broader behavioral patterns. Kunutsor and Laukkanen (2023) reported interactions between sauna use, physical activity, inflammatory markers, and socioeconomic status, indicating that associations may not be attributable to thermal exposure alone [72].

Overall, current evidence does not support conclusions regarding universal safety or independent preventive efficacy, and further standardized longitudinal research is required.

### 4.9. Limitations and Future Directions

Several limitations of this review should be considered.

The literature search was confined to two databases. Although these sources cover a substantial portion of medical publications, relevant studies indexed elsewhere or unpublished data may not have been captured. In addition, this review was conducted as a narrative synthesis rather than a systematic review or meta-analysis, and no formal assessment of methodological quality or risk of bias was undertaken.

The available evidence is geographically concentrated. Most studies on habitual bathtub bathing were conducted in Japan, whereas long-term outcome data for sauna bathing originate predominantly from Finland. Because bathing practices are shaped by climate, housing environment, and cultural norms, the generalizability of these findings to other populations remains uncertain.

Definitions of exposure varied across studies. Frequency, temperature, session duration, immersion depth, and humidity were not consistently reported, and many investigations relied on self-reported questionnaires. Such variability makes direct comparison difficult and may introduce measurement error. The heterogeneity of exposure definitions also makes it difficult to interpret the findings in terms of a clear dose–response relationship, particularly for household bathing practices.

Much of the evidence for habitual bathtub bathing is based on cross-sectional analyses, which restrict causal interpretation and leave room for residual confounding. Differences in overall health behavior and socioeconomic background may influence both bathing habits and health outcomes, and reverse causation cannot be excluded.

Several publications are based on the same participant cohort and intervention protocol; therefore, the apparent number of studies may overrepresent evidence from a single population. Careful interpretation is required when considering the cumulative strength of evidence.

Finally, interventional studies of hot-water immersion and sauna therapy have generally been small and short in duration. Younger populations remain underrepresented, and standardized protocols assessing long-term clinical outcomes are lacking.

Further research with clearly defined exposure parameters, objective assessment of thermal load, and long-term follow-up will be necessary to clarify the role of habitual thermal practices in cardiovascular prevention.

## 5. Conclusions

The present review summarized current evidence on habitual bathtub bathing and sauna use in relation to vascular and cardiometabolic health.

Prospective data, largely from Finnish cohorts, indicate that frequent sauna bathing is associated with lower cardiovascular and all-cause mortality, although these findings are based on a limited number of populations. In contrast, research on domestic bathtub bathing has focused mainly on observational studies assessing surrogate markers, and its association with incident ASCVD events has not yet been evaluated.

Thermal exposure produces acute circulatory and molecular responses that are relevant to cardiovascular regulation. Whether repeated exposure translates into long-term cardiovascular benefit remains to be clarified. Continued investigation with clearly defined exposure parameters and clinical endpoints will be important to better understand the role of habitual thermal practices in cardiovascular health.

## Figures and Tables

**Table 1:** Characteristics of studies on habitual bathtub bathing and related health outcomes.

References	Study Design	Sample (*n*, Age)	Country	Exposure (Freq; Temp; Time)	Effect on Outcome(s)
[5] Miyata et al. (2023)	Cross-sectional (community health examination cohort)	*n* = 202 (≥65 yrs); *n* = 524 with CAVI	Japan, Kagoshima	Daily bathing: 80.3%; not daily: 19.7%; Onsen use ≥1 time/week: 44.8%; <1 time/week: 34.7%	No assoc. with sleep; Daily bathing linked to ↓ CAVI; Onsen freq. not assoc. with CAVI
[7] Kohara et al. (2015)	Cross-sectional	*n* = 875 (adults at Anti-aging/Preventive Med Center)	Japan, Ehime	Bathing freq: 7/wk 51.5%, 5–6/wk 22.4%, ≤4/wk 26.1%; Temp: >42 °C 15.5%, 39–42 °C 77.8%, <39 °C 3.6%	↑ bathing freq associated with ↓ carotid IMT, ↓ baPWV, ↓ BNP, ↑ calcaneal bone transmission speed; Higher temp linked to ↓ baPWV. baPWV: ↓ with higher bathing temperature
[12] Kohara et al. (2018)	Cohort (longitudinal)	*n* = 873; community-dwelling older adults, mean age ~72 yrs	Japan	Bathing frequency: Group I (0–4 times/wk) 26.1%, Group II (5–6 times/wk) 22.7%, Group III (>7 times/wk) 51.2%; Bathing duration 0–120 min (mean 12.4 ± 9.9 min); Water temperature: hot (>41 °C), medium (40–41 °C), lukewarm (<40 °C). Higher freq.: significantly lower mean age. Higher freq.: ↑ temp, ↓ duration (sig.)	↑ bathing freq (≥5/wk) assoc. with ↓ baPWV, ↓ central PP, ↓ BNP; Higher bath temp linked to ↓ baPWV; Longitudinal follow-up showed frequent bathing suppressed ↑ BNP and high temp bathing tended to suppress progression of baPWV & carotid IMT; No clear assoc. with bath duration
[16] Takamura et al. (2010)	Cross-sectional (exploratory)	*n* = 10 young men (mean 20.6 yrs); *n* = 11 older men (mean 77.2 yrs)	Japan, Koshinetsu region (Yamanashi, Nagano, Niigata)	Young: mainly shower, avg tub bathing 0.9 times/wk; Older: avg tub bathing 5.7 times/wk; Temp: both groups preferred warm–hot; Bath duration longer in older adults	Older men: ↑ CAVI with higher bathing freq & onsen use; Younger men: exercise habit linked to preference for lower temp; Comfort & fatigue reduction differed by age and bath temp; No clear assoc. with sleep
[17] Iiyama et al. (2022)	Cross-sectional	*n* = 60 (34 men, 26 women)	Japan, Kumamoto	Thermal habit group: ≥5 days/week (39 participants, 65%) with immersion at 40–41 °C for 10 min or 39 °C for 20 min; Non-habit group: shower only or 1–2 baths/week (18 participants, 30%)	No significant differences in lifestyle profiles between groups
[18] Ishizawa et al. (2012)	Cross-sectional	*n* = 198 (healthy adults, men & women)	Japan	Bathing frequency: 67.7% bathed daily (7 days/week); most common water temperature 40–41 °C (40.2%), mean 40.1 ± 1.1 °C; most common duration 10–15 min (30.9%), mean 11.9 ± 9.0 min; 83.3% practiced full-body immersion, 33.3% immersed up to shoulders, 55.0% immersed with shoulders exposed	↑ bathing freq assoc. with ↓ tension–anxiety, ↓ depression, ↑subjective health; Frequent bath additive use assoc. with ↑subjective health & sleep quality
[19] Tai et al. (2021)	Cohort (longitudinal)	*n* = 1094; community-dwelling older adults, mean age ~72 yrs	Japan, Nara	Bathing before bedtime: 0–60 min, 61–120 min, or 121–180 min prior to sleep; average duration ~15 min; frequency: ~30% bathed within 1 h before sleep; water temperature: NR	Bathing 0–60 min before bedtime most strongly assoc. with ↓ sleep onset latency; Bathing 61–180 min also linked to ↓ sleep onset latency (actigraphic & self-reported) and ↑ distal-to-proximal skin temp gradient (DPG); ↑ DPG assoc. with shorter sleep latency
[20] Yamasaki et al. (2022)	Cross-sectional	*n* = 10,428; community-dwelling older adults ≥65 yrs	Japan, Oita	Frequency of hot spring bathing: daily 44.7%, 4–6 times/week 20.6%, 1–3 times/week 15.7%, 1–3 times/month 10.9%, seldom 8.1%; bathing duration: <10 min 4.1%, 10–19 min 35.5%, 20–29 min 30.7%, 30–39 min 15.9%, ≥40 min 13.8%; bathing history: <10 yrs 20.0%, 10–19 yrs 21.6%, 20–29 yrs 20.3%, 30–39 yrs 16.3%	Nighttime hot spring bathing (≥19:00) assoc. with ↓ hypertension (OR 0.85, 95% CI 0.77–0.94); Daily bathing and 10–29 min duration also linked to ↓ hypertension
[21] Itoh et al. (2021)	Randomized crossover trial	*n* = 11; healthy men, mean age 45.6 yrs	Japan	Group A: shower 40 °C, 10 min daily for 5 days; Group B: full bath 40 °C, 10 min daily for 5 days; 1 month later groups crossed over; all underwent test bath 40 °C, 15 min full bath with warming	Full bath vs. shower: ↑ core body temp, ↑ HSP70, ↓ fatigue & muscle pain (VAS), ↑ mood (↓ POMS confusion)
[22] Ishizawa et al. (2018)	Interventional	*n* = 12; healthy adults, mean age ~22 yrs	Japan	Single full-body immersion bath at 40 °C for 15 min	Bathing assoc. with ↑ core body temp, ↓ salivary amylase activity, ↑ relaxation & mood state
[23] Ishikawa (2014)	Randomized crossover trial	*n* = 28; hypertensive patients (13 men, 15 women), mean age 65.5 yrs	Japan	Bathing vs. non-bathing day during 24 h ambulatory BP monitoring; order randomized (A: bathing → non-bathing, B: non-bathing → bathing); bathing duration ~33.6 ± 13.6 min; water temp ~40.6 ± 1.5 °C	Bathing assoc. with ↓ brachial & central BP (due to ↓ reflection wave); No change in PWV
[24] Ohgomori (2021)	Crossover trial	*n* = 16; healthy adults, mean age ~21 yrs	Japan	Bathing in warm water at 40 °C, shoulder immersion for ≥10 min, between 18:00–24:00; Shower condition at ~40 °C as control; Intervention period 2 weeks	Bathing assoc. with ↑ prefrontal cortex blood flow (NIRS) and ↑ cognitive performance vs. control
[25] James TJ et al. (2023) *	Pre–post-intervention study (14 days)	*n* = 14; T2DM; ≥35 y (mean 65 ± 8); male 57%	UK	8–10 sessions/14 days (≤1/day); 40 °C; 60 min; target Trec 38.5–39.0 °C	↑ fasting insulin sensitivity (QUICKI; *p* = 0.03); ↓ fasting insulin (*p* = 0.04); fasting glucose ns; postprandial insulin sensitivity/glucose/insulin ns; eHSP70 and IL-6/IL-10/TNF-α ns; ↓ RMR (−6.6%, *p* = 0.045); ↓ resting VO_2_ (*p* = 0.05); CHO/fat oxidation ns.
[26] James et al. (2024) *	Pre–post-intervention study (14 days)	*n* = 14; T2DM; ≥35 y (mean 65 ± 8); mele 57%	UK	8–10 sessions/14 days (≤1/day); 40 °C; 60 min; target Trec 38.5–39.0 °C	Macrovascular endothelial function (brachial FMD) ns (*p* = 0.43); cutaneous microvascular endothelial function (ACh/insulin iontophoresis) ns; NOX ns (*p* = 0.38); ↓ SBP (Δ − 9 mmHg, *p* = 0.03); DBP ns (*p* = 0.58); ↓ resting HR (*p* = 0.01); ↓ stroke volume index (*p* = 0.02); ↓ cardiac index (*p* = 0.01).
[27] Hoekstra et al. (2018)	Randomized crossover trial (acute) + interventional (controlled, 2-wk; pre–post)	INT *n* = 10 sedentary overweight men (BMI 31.0 ± 4.2; age 33.2 ± 10.1); CON *n* = 8 BMI-matched men (BMI 30.0 ± 2.5; age 32.0 ± 7.5)	UK	Acute: HWI 39 °C, 1 h (neck immersion) vs. AMB 1 h; blood pre/post/post + 2 h. Chronic: 10 HWI sessions/2 wk (39 °C; first 5 × 45 min, last 5 × 60 min; neck immersion) vs. CON (resting blood matched time points).	Acute (HWI vs. AMB): ↑ IL-6 (*p* < 0.001); ↑ nitrite (*p* = 0.04); iHsp72 ns (*p* = 0.57). Chronic (INT vs. CON): ↓ fasting glucose (*p* = 0.04); ↓fasting insulin (*p* = 0.04); ↓ eHsp72 (*p* = 0.03); resting iHsp72 ns (*p* = 0.59).
[28] Roxburgh et al. (2023)	Randomized controlled trial (parallel, multi-arm; 12-week intervention; acute and adaptive analyses)	Heat *n* = 27; HIIT *n* = 25; Home *n* = 26; severe lower-limb osteoarthritis; mean age 66–71 yrs	New Zealand	Heat: 3 sessions/week for 12 weeks; 39.9–40.0 °C water immersion (mid-sternal level) 20–30 min; followed by 10–20 min light resistance exercise	Acute (Heat): ↓ SBP (−10–12 mmHg), ↓ DBP (−4–7 mmHg), ↓ MAP (−8–13 mmHg) during and post-immersion (20 min recovery); ↑ HR (~+16 bpm); ↑ core temp (+0.9 °C). Chronic (12 wk): ↓ resting SBP (−9 mmHg, *p* < 0.001); ↓ DBP (−4 mmHg, *p* ≤ 0.001); ↓ MAP (−6 mmHg); no change in HR or HRV; no improvement in HbA1c or CGM-derived glycemic indices.
[29] Akerman et al. (2019)	Randomized controlled trial (parallel-group; 12-wk intervention)	Heat *n* = 11; Exercise *n* = 11; PAD (Fontaine IIa–IIb); mean age 74–76 yr	New Zealand	Spa bathing ~39 °C; 3–5 days/week; 20–30 min immersion (shoulder depth) + ≤30 min calisthenics; 12 weeks	↑ 6MWT maximum walking distance (+41 m, *p* = 0.006); ↑ pain-free walking distance (+43 m, *p* < 0.001); ↓ SBP (−7 mmHg heat vs. −3 mmHg exercise; interaction *p* = 0.049); ↓ DBP (−4 mmHg, *p* = 0.002); ↓ MAP (−4 mmHg, *p* < 0.001); VEGF ↑60% (*p* = 0.011); ABI ns; FMD ns; PWV ns; blood volume ns; limb blood flow (VOP) ns.

Exposure variables (frequency, temperature, duration) are summarized as reported in each study. NR (not reported) indicates variables not available in the original article. Reported effects represent the primary outcomes described by the authors. ns indicates “not significant.” Arrows: ↑ = increase/improvement, ↓ = decrease/reduction. References are numbered according to the final reference list (to be updated at the proof stage). * These papers report different outcomes from the same hot-water immersion intervention cohort (*n* = 14; pre–post design; 8–10 sessions over 14 days). Findings should be interpreted as derived from a single study population.

**Table 2:** Characteristics and main findings of studies on sauna bathing habits and health outcomes.

References	Study Design	Sample (*n*, Age, Sex)	Country/Region	Sauna Modality	Exposure (Freq; Temp; Time; Humidity)	Effect on Outcome(s)
[30] Laukkanen et al. (2015) †	Prospective cohort (KIHD; median follow-up 20.7 y)	*n* = 2315; men; 42–60 y (mean 53.1)	Eastern Finland (Kuopio region)	Traditional Finnish sauna	Freq: 1, 2–3, 4–7 sessions/week (25.96%, 65.36%, 8.68%); Duration: <11, 11–19, ≥19 min; Temp: NR; Humidity: NR	4–7/wk vs. 1/wk → ↓ SCD (HR 0.37, 95% CI 0.18–0.75); ≥19 vs. <11 min → ↓ SCD (HR 0.48, 0.31–0.75); also ↓ fatal CHD/CVD & ↓ all-cause mortality
[31] Kunutsor et al. (2024a) †	Prospective cohort (KIHD; median follow-up 27.8 y)	*n* = 2575; men; 42–61 y (mean ~53)	Eastern Finland (Kuopio region)	Traditional Finnish sauna	Freq: 0–7/wk (median 2/wk; IQR 1–2); Session: median 10 min (IQR 8–15); Temp: mean 78.9 °C (SD 9.6); Humidity: NR	High SBP (≥140 mmHg) → ↑ all-cause mortality; higher sauna frequency → ↓ all-cause mortality; interaction: frequent sauna attenuated the excess mortality risk associated with high SBP; highest risk observed in high SBP + low sauna frequency.
[32] Laukkanen et al. (2023) †	Prospective cohort (KIHD; median follow-up 27.8 y)	*n* = 2575; men; 42–61 y (baseline mean ~53)	Eastern Finland (Kuopio region)	Traditional Finnish sauna	FSB: ≤2 vs. 3–7/wk; SBP strata (≥130/≥140 mmHg); Temp: NR; Duration: NR; Humidity: NR	SBP ≥ 140 → ↑ CVD mortality (HR 1.44, 1.23–1.68); high FSB (3–7/wk) → ↓ CVD mortality (HR 0.81, 0.68–0.97); interaction: high SBP + low FSB → highest risk (HR 1.81, 1.39–2.36); high SBP + high FSB → attenuated (HR 1.52, 1.06–2.16).
[33] Laukkanen et al. (2018c) †	Prospective cohort (KIHD; median follow-up 15.0 y)	*n* = 1688; men 821 (48.6%), women 867 (51.4%); 53–74 y (mean 63)	Eastern Finland (Kuopio region)	Traditional Finnish sauna	Freq: 1, 2–3, 4–7/wk (26.95%, 60.9%, 12.14%; median 2 [IQR 1–3]); Temp: 75.9 ± 9.9 °C; Time: 30 min/wk (median 15–45); Humidity: NR	Effect: 4–7 vs. 1/wk → ↓ CVD mortality (HR 0.30, 0.14–0.64); 2–3 vs. 1/wk → HR 0.71 (0.52–0.98). ≥45 vs. ≤15 min/wk → ↓ CVD mortality (HR 0.49, 0.30–0.80). Risk prediction improved (C-index + 0.0091, NRI 4.14%).
[34] Kunutsor et al. (2022a) †	Prospective cohort (KIHD; median follow-up 27.8 y)	*n* = 2681; men; 42–61 y (mean 53)	Eastern Finland (Kuopio region)	Traditional Finnish sauna	Freq: ≤1/wk, 2–3/wk, 4–7/wk (median 2/wk); Temp: NR; Duration: NR; Humidity: NR	4–7/wk vs. ≤1/wk → ↓ all-cause mortality; FSB inversely assoc. with hsCRP (r = −0.06, *p* < 0.001).
[35] Kunutsor et al. (2018a) †	Prospective cohort (KIHD; median follow-up 14.9 y)	*n* = 1628; men 788, women 840; 53–74 y (mean 62.7)	Eastern Finland (Kuopio & surrounding rural areas)	Traditional Finnish sauna	Freq: 1, 2–3, 4–7/wk (26.7%, 61.9%, 12.1%); Temp: NR; Duration: NR; Humidity: NR	4–7/wk vs. 1/wk → ↓ stroke (HR 0.38, 95% CI 0.18–0.81); ↓ ischemic stroke (HR 0.42, 0.18–0.96); hemorrhagic stroke ns (HR 0.33, 0.07–1.51); 2–3/wk vs. 1/wk ns
[36] Kunutsor et al. (2017a) †	Prospective cohort (KIHD; median follow-up 25.6 y)	*n* = 1935; men; 42–61 y; no prior respiratory disease	Eastern Finland (Kuopio region)	Traditional Finnish sauna	Freq: ≤1/wk 25.89%, 2–3/wk 65.22%, ≥4/wk 8.89% (median 2/wk; IQR 1–3); Session: mean 14.0 min (SD 7.2); Temp: NR; Humidity: NR	2–3/wk vs. ≤1/wk → ↓ respiratory diseases (COPD/asthma/pneumonia) (adj. HR 0.68, 95% CI 0.55–0.86); ≥4/wk vs. ≤1/wk → ↓ risk (adj. HR 0.53, 0.34–0.84); pneumonia subset similar (e.g., 2–3/wk HR 0.72, 0.57–0.90).
[37] Laukkanen et al. (2018b) †	Prospective cohort (KIHD; median follow-up 24.9 y)	*n* = 2138; men; 42–61 y; no prior psychotic disorder	Eastern Finland (Kuopio region)	Traditional Finnish sauna	Freq: 1/wk 25.12%, 2–3/wk 66.28%, 4–7/wk 8.61%; Temp: NR; Duration: NR; Humidity: NR	4–7/wk vs. 1/wk → ↓ psychotic disorders (multivariable HR 0.21, 95% CI 0.08–0.52); result robust to additional adjustments.
[38] Kunutsor et al. (2018b) †	Prospective cohort (KIHD; 11 y follow-up)	*n* = 2269; men; 42–61 y	Eastern Finland (Kuopio region)	Traditional Finnish sauna	Freq: 1, 2–3, 4–7/wk (26.2%, 65.2%, 8.6%); Temp: ~80–100 °C; Time: 5–20 min; Humidity: 10–20%	Higher sauna freq → ↓ hsCRP, ↓ fibrinogen, ↓ WBC (baseline & follow-up). No assoc. with oxidative stress marker (GGT).
[39] Kunutsor et al. (2023) †	Prospective cohort (KIHD; median follow-up 25.7 y)	*n* = 2071; men; 42–61 y; normal renal function at baseline	Eastern Finland (Kuopio region)	Traditional Finnish sauna	Freq: 1, 2–3, 4–7/wk; Temp: NR; Duration: NR; Humidity: NR	CKD incidence: ns across frequency groups; eGFR/serum creatinine/Na: ns; serum K^+^: ↑ +0.05 mmol/L (*p* = 0.033); also ↓ all-cause mortality with higher frequency (HR ~0.78; *p*≈0.03).
[40] Laukkanen et al. (2018a) †	Prospective cohort (KIHD; median follow-up 26.1 y)	*n* = 2291; men; 42–61 y (baseline)	Eastern Finland (Kuopio region)	Traditional Finnish sauna	FSB: low ≤ 2 vs. high 3–7/wk; CRF: low vs. high (median cutoffs); Temp: mean ~79 °C; Duration: NR; Humidity: NR	High CRF and high FSB each assoc. with ↓ SCD; combined high CRF + high FSB → lowest SCD; either alone high → ↓ SCD.
[41] Kunutsor et al. (2024b) †	Prospective cohort (KIHD; 11 y follow-up)	*n* = 2012; men; 42–61 y	Eastern Finland (Kuopio region)	Traditional Finnish sauna	Freq: 1, 2–3, 4–7/wk (26.4%, 65.4%, 8.3%); Time: NR; Temp: NR; Humidity: NR	Higher sauna freq → ↑ baseline CRF (r = 0.07, *p* = 0.002). Longitudinally, 2–3/wk (not 4–7/wk) → small ↑ CRF over 11 y (+1.22 mL/kg/min, *p* = 0.038). Session duration not assoc. with CRF.
[42] Kunutsor et al. (2017b) †	Prospective cohort (KIHD; median follow-up 25.6 y)	*n* = 2210; men; 42–61 y	Eastern Finland (Kuopio region)	Traditional Finnish sauna	Freq: median 2/wk (range 0–7); Temp: mean 78.9 °C (SD 9.6); Time: NR; Humidity: NR	Higher sauna freq → ↓ pneumonia risk. 2–3/wk vs. ≤1/wk: HR 0.72 (95% CI 0.57–0.90); 4–7/wk vs. ≤1/wk: HR 0.63 (0.39–1.00).
[43] Laukkanen et al. (2019) †	Prospective cohort (KIHD; follow-up up to 29 y)	*n* = 2173; men; 42–61 y at baseline	Eastern Finland (Kuopio region)	Traditional Finnish sauna	Freq: ≤1, 2–3, ≥4/wk (25.9%, 65.2%, 8.9%); Temp: ~80–100 °C; Time: 5–20 min; Humidity: 10–20%	No significant assoc. between sauna freq and overall cancer risk or site-specific cancers (prostate, GI). Possible ↓ lung cancer risk in subset analyses, but not statistically robust.
[44] Kunutsor et al. (2019) †	Prospective cohort (KIHD; median follow-up 24.9 y)	*n* = 2242; men; 42–61 y; free of prior VTE	Eastern Finland (Kuopio region)	Traditional Finnish sauna	Freq: ≤1/wk, 2–3/wk, ≥4/wk (median 2/wk); Temp: NR; Duration: NR; Humidity: NR	2–3/wk vs. ≤1/wk → ↓ VTE (HR 0.67, 95% CI 0.46–0.96); ≥4/wk vs. ≤1/wk ns (HR 0.92, 0.51–1.68); most reduction at 2–3/wk.
[45] Kunutsor & Laukkanen (2021) †	Prospective cohort (KIHD; median follow-up 26.6 y)	*n* = 2210; men; 42–61 y (mean 53)	Eastern Finland (Kuopio region)	Traditional Finnish sauna	Freq: 1, 2–3, 4–7/wk; Temp: NR; Time: NR; Humidity: NR	High CRF and frequent sauna each → ↓ pneumonia risk. 4–7 vs. 1/wk: HR 0.59 (95% CI 0.43–0.81). Combination of high CRF + frequent sauna → greatest reduction.
[46] Kunutsor et al. (2022c) †	Prospective cohort (KIHD; median follow-up 26.6 y)	*n* = 2264; men; 42–61 y	Eastern Finland (Kuopio region)	Traditional Finnish sauna	Median freq: 2/wk (range 0–7). Distribution: ≤1/wk 35.3%, 2–7/wk 64.7%. Temp: NR; Time: NR; Humidity: NR	hsCRP ↑ → ↑ pneumonia risk (HR 1.30). Frequent sauna (2–7/wk) → ↓ pneumonia risk (HR 0.79). hsCRP high + low sauna → highest risk (HR 1.67). hsCRP high + frequent sauna → risk attenuated (HR 0.94).
[47] Kunutsor et al. (2022b) †	Prospective cohort (KIHD; median follow-up 27.8 y)	*n* = 2575; men; 42–61 y (baseline)	Eastern Finland (Kuopio region)	Traditional Finnish sauna	FSB (self-reported): ≤2 vs. 3–7/wk (median 2/wk; IQR 1–2); Temp: NR; Duration: NR; Humidity: NR	Low SES → ↑ all-cause mortality (HR 1.31, 95% CI 1.18–1.45); high FSB (3–7/wk) → ↓ all-cause mortality (HR 0.86, 0.76–0.97); interaction: low SES + low FSB → highest risk (HR 1.35, 1.20–1.51); low SES + high FSB → attenuated (HR 1.07, 0.89–1.29).
[48] Zaccardi et al. (2017) †	Prospective cohort (KIHD; median follow-up 24.7 y)	*n* = 1621 men; 42–60 y; free of hypertension at baseline	Finland (Eastern Finland)	Traditional Finnish sauna	Freq: 1/wk (ref), 2–3/wk, 4–7/wk; Temp: ~79–81 °C; Duration: mean 14.4 min/session; Humidity: dry (NR)	4–7/wk → ↓ incident hypertension (HR 0.53, 95% CI 0.28–0.98); 2–3/wk ns after full adjustment (HR 0.83, 95% CI 0.59–1.18)
[49] Kunutsor et al. (2018) †	Prospective cohort (KIHD; median follow-up 26.1 y)	*n* = 2277 men; 42–61 y	Finland (Eastern Finland)	Traditional Finnish sauna	Sauna frequency: ≤2 vs. 3–7 sessions/week; Temp: 80–100 °C (traditional Finnish sauna; dry air 10–20%); Time: NR (session duration	High vs. low FSB: ↓ CVD mortality HR 0.74 (0.59–0.94); ↓ all-cause mortality HR 0.84 (0.72–0.97). Joint: (ref = low CRF & low FSB) high CRF & high FSB → ↓ CVD mortality HR 0.42 (0.28–0.62); ↓ all-cause HR 0.60 (0.48–0.76).
[50] Pöyhönen et al. (2022)	Prospective cohort (TAMUS; baseline 1994, follow-ups 1999 & 2004)	*n* = 1306; men; middle-aged & older	Tampere region, Finland	Traditional Finnish sauna *	Freq: 0–1/wk 39.7%, 2/wk 43.0%, ≥3/wk 17.3%; Temp: NR; Duration: NR; Humidity: NR	No clear assoc. with overall LUTS; ≥3/wk → ↓ feeling of incomplete emptying (vs. 0–1/wk); other LUTS ns
[51] Strandberg et al. (2018)	Cross-sectional (HBS; 2015 survey)	*n* = 524; men; mean 86 y (range 80–95)	Finland (Helsinki)	Traditional Finnish sauna *	57.6% year-round users (*n* = 302); 17.6% seasonal only (*n* = 92); 24.8% no current sauna use (92.2% of them had prior use). Most 1/wk, ~10% ≥2/wk. Temp: ~80 °C; Time: ~15 min; Humidity: NR.	Current users → ↑ HRQoL (physical function, vitality, social function, general health). Non-users more often had HF, musculoskeletal disorders, mobility limitations.
[52] Engström et al. (2024)	Cross-sectional (MONICA 2022)	*n* = 971 answered (of 1180); adults 25–74 y; sauna users *n* = 641 (66%)	Northern Sweden (Norrbotten & Västerbotten)	Mixed; mainly traditional	Users vs. non-users; Frequency: <1/wk 46%, 1/wk 28%, 2–3/wk 23%, ≥4/wk 3%; Session: 15–20 min, 1–2 rounds; Temp: 60–80 °C; Humidity: NR	Users → ↓ diagnosed hypertension, ↓ self-reported pain, ↑ wellbeing/vitality, ↑ sleep satisfaction, ↑ general & mental health (cross-sectional; assoc.)
[53] Gravel et al. (2021)	Experimental pre–post, single-group clinical intervention (acute effects)	*n* = 22; men 20, women 2; mean age 67 ± 10 y; stable CAD with prior PCI/CABG)	Montréal, Canada	Traditional Finnish sauna	Freq: single session (2 bouts); Temp: 81.3 ± 2.7 °C; Time: 10 min ×2 with 10 min ambient rest; Humidity: 23 ± 3%	Core temp ↑ (+0.66 °C, *p* < 0.01), HR ↑ (+27 bpm, *p* < 0.01), SBP ↓ (−19 mmHg, *p* < 0.01), DBP ↓ (−6 mmHg, *p* < 0.01), brachial FMD ↑ (+1.21%, *p* = 0.04); PORH ns; IL-6 ↑ (*p* < 0.01); other inflammatory markers ns.
[54] Hussain et al. (2019)	Cross-sectional (online survey)	*n* = 482; adults 17–80 y (mean 45); 48.7% women	29 countries (mainly Finland 28.4%, Australia 25.3%, USA 20.5%)	Traditional sauna (Finnish, Russian, etc.) most common (73% of respondents); also steam (22%), dry (22%), far-infrared (20%), wet (12%), Japanese bath type (6%), others (7%). Multiple responses allowed.	Median freq: 6/mo (IQR 4–12). Session: median 16 min (range 5–90). Total duration: median 49 min. Temp: most common 80 °C. Cooling: shower 66.9%, cold water/air ≈45%. Drinks: water 63.7%, alcohol 31.6%. Humidity: NR.	Reported outcomes: ↑ relaxation/stress relief (100%), ↑ vitality (99.6%), ↑ sleep quality (83.5%), ↓ pain (87.8%); 67.4% of those with illness improved. Adverse effects: dizziness (49.2%), dehydration (40.1%), headache (23.1%); severe AEs rare (0.8%).
[55] Lee E, Kolunsarka I et al. (2022)	Randomized controlled trial (8 wk)	*n* = 47; 42 women, 6 men; 30–64 y (mean 49 ± 9); sedentary adults with ≥1 CVD risk factor	Finland (Jyväskylä)	Traditional Finnish sauna (post-exercise)	3 sessions/week; 15 min; 65–75 °C; 10–20% humidity; 8 weeks	Exercise + sauna vs. exercise alone → ↑ CRF (Δ + 2.7 mL/kg/min); ↓ SBP (−8.0 mmHg); ↓ total cholesterol (−19 mg/dL); DBP, PWV, AIx ns.
[56] Miyamoto H, Kai H et al. (2005)	Prospective single-arm interventional study (4-week repeated sauna therapy)	*n* = 15; 12 men, 3 women; mean age 62 ± 15 y; chronic systolic CHF (NYHA class 2.8 ± 0.4)	Japan (Kurume)	60 °C dry sauna (medical thermal therapy protocol)	60 °C; 15–20 min/session; then 30 min rest at 25 °C with warming; once/day, 5 days/week; 4 weeks; humidity NR	No adverse events; ↓ SBP (101 ± 13 → 98 ± 14 mmHg); ↓ rate–pressure product; HR ns; ↑ LVEF (30 ± 11 → 34 ± 11%); ↑ 6 min walk distance (388 ± 110 → 448 ± 118 m); ↑ peak VO_2_ (13.3 ± 1.8 → 16.3 ± 2.7 mL/kg/min); ↑ anaerobic threshold (9.4 ± 1.2 → 11.5 ± 1.9 mL/kg/min); ↓ epinephrine; ↓ norepinephrine; BNP ns; During 12-month follow-up: 2/15 CHF deaths reported; ↓ hospital admissions for CHF (compared with prior year).
[57] Basford et al. (2008)	Randomized controlled cross-over pilot trial (4-week intervention; no washout)	*n* = 9; chronic CHF; NYHA III–IV; LVEF 20.0 ± 6.9%; mean age 71.6 ± 9.8 yr	USA	Dry sauna (60 °C, supervised medical protocol)	60 ± 1 °C dry sauna; 3 sessions/week; 15–20 min/session + 30 min supine rest; 4 weeks	No adverse events; Resting HR ↓ (*p* = 0.02); Noradrenaline ↓ 24% (*p* = 0.049; between-group significant); Endothelin ↓ (within-group *p* < 0.0039); MLWHFQ improved vs. baseline (between-group ns); Treadmill endurance ns; No deterioration in BP or ECG.

* = The sauna style was not specified in Methods but described in the Introduction as typical Finnish sauna bathing. Exposure variables (frequency, temperature, duration, humidity) were marked as NR (not reported) when not available in the original article. Reported effects represent the primary outcomes described in each study. ns indicates “not significant.” Arrows: ↑ = increase, ↓ = decrease. † Studies derived from the Kuopio Ischaemic Heart Disease (KIHD) cohort. These reports represent different outcome analyses from the same underlying population.

## Data Availability

No new data were created or analyzed in this study. Data sharing is not applicable to this article.

## Abbreviations

The following abbreviations are used in this manuscript:

ANP	Atrial natriuretic peptide
ASCVD	Atherosclerotic cardiovascular disease
baPWV	Brachial-ankle pulse wave velocity
BMI	Body mass index
BNP	B-type natriuretic peptide
BP	Blood pressure
CAD	Coronary artery disease
CAVI	Cardio-ankle vascular index
CHD	Coronary heart disease
CHF	Chronic heart failure
CI	Confidence interval
CKD	Chronic kidney disease
COPD	Chronic obstructive pulmonary disease
CRF	Cardiorespiratory fitness
CVD	Cardiovascular disease
DBP	Diastolic blood pressure
DPG	Distal-to-proximal skin temperature gradient
FMD	Flow-mediated dilation
FSB	Frequency of sauna bathing
HBS	Helsinki Businessmen Study
HR	Hazard ratio
hsCRP	High-sensitivity C-reactive protein
HSP	Heat shock protein
HSP70	Heat shock protein 70
HWI	Hot-water immersion
IL-6	Interleukin-6
IMT	Intima-media thickness
IQR	Interquartile range
KIHD	Kuopio Ischaemic Heart Disease Risk Factor Study
LUTS	Lower urinary tract symptoms
LVEF	Left ventricular ejection fraction
MAP	Mean arterial pressure
NYHA	New York Heart Association functional classification
OR	Odds ratio
POMS	Profile of Mood States
SBP	Systolic blood pressure
SCD	Sudden cardiac death
SES	Socioeconomic status
T2DM	Type 2 diabetes mellitus
TAMUS	Tampere Ageing Male Urologic Study
VTE	Venous thromboembolism
